# How prior experience, cognitive skills and practice are related with eye-hand span and performance in video gaming

**DOI:** 10.16910/jemr.11.3.1

**Published:** 2018-05-11

**Authors:** Markus Nivala, Agnes Cichy, Hans Gruber

**Affiliations:** Department of Education, Communication and Learning, University of Gothenburg, Sweden; Department of Educational Science, University of Regensburg, Germany; Department of Educational Science, University of Regensburg, Germany; Department of Teacher Education, University of Turku, Finland

**Keywords:** Cognitive skills, eye movement, eye-hand span, performance, practice, prior experience, video gaming, eye tracking, new media, gaze

## Abstract

Research has shown that performance in visual domains depends on domain-specific cognitive and perceptual adaptations that result from extensive practice. However, less is known about processes and factors that underpin the acquisition of such adaptations. The present study investigated how prior experience, cognitive skills, task difficulty and practice effect eye-hand span (EHS) and performance in video gaming. Thirty-three participants played a platformer video game in a pre-test/practice/post-test experiment. Eye movements and keypresses were recorded. The results show that a short practice period improved performance but did not increase EHS. Instead, EHS was related to task difficulty. Furthermore, while EHS correlated with initial performance, this effect seemed to diminish after practice. Cognitive skills (concentration endurance, working memory, mental flexibility and executive functioning) predicted performance in some parts of the experiment. The study offers insights into the early development of visual adaptations and performance.

## Introduction

Research on expertise has shown that performance in visual domains such as music, chess, sports, and medicine depends on domain-specific cognitive and perceptual adaptations ( 7, 11, 16 ). However, less is known about processes that underpin the acquisition of such adaptations and about how prior experience and general perceptual and cognitive skills affect the development of performance ( 31 ). Furthermore, due to the domain-specific nature of these adaptations, comparisons across domains are rare. Yet, as Ericsson and Smith ( 8 )argue, “[t]he most effective approach to organizing the results across domains of expertise is to propose a small number of learning mechanisms that can account for the development of similar performance characteristics in different domains” (p. 32). Therefore, the present study investigated how prior experience, cognitive skills, task difficulty and practice effect the novices’ eye-hand span (EHS) and performance in a simple jump-and-run (platformer) video game. The study explicitly tries to relate the findings to findings from the domain of music in which eye-hand span and its relation to performance has been studied more extensively.

Research on musicians’ expertise in sight-reading offers a particularly interesting parallel to the present study. Both jump-and-run platformer games and sight-reading require scanning of the incoming information and precise timing of motor actions ( 24 ). Furthermore, in both domains, visual information has to be processed with constant speed (tempo) and is presented sequentially (information is processed in order from left to right). However, the domains also have considerable differences. Sight-reading relies on canonical forms of visual information (notation) and accomplished musicians have years of experience in interpreting it. In contrast, video games in general offer a multitude of highly varied visual and motor tasks. Therefore, prior experience in video gaming is not directly comparable to musicians’ prior experience in sightreading.

In music, eye-hand span is commonly expressed either in milliseconds, notes or beats between the eye position and the currently played notes. For example, Truitt, Clifton, Pollatsek, and Rayner ( 30 ) found that skilled musicians have a larger EHS. On the other hand, Furneaux and Land ( 10 )found that while professionals showed less variation in EHS, both professionals and amateurs had an EHS of approximately one second, but that EHS varied with playing tempo. However, when measured in notes (number of notes between the hand and eye position), professionals had a larger EHS of approximately four notes, whereas for amateurs it was two. This led Furneaux and Land ( 10 ) to conclude that while professionals have larger information buffers, EHS is rather related to playing tempo than expertise. Similarly, Penttinen, Huovinen, and Ylitalo ( 22 ) found that EHS decreased when the participants encountered an unexpected melodic alteration. In brief, EHS seems to be related to the musicians’ skill level and task difficulty, but the details of this relation are unclear.

In a recent study, Rosemann et al. ( 24 )studied how practice, playing tempo, musical complexity, and cognitive skills influence the size of EHS of pianists. While not a direct replication, the current study was originally inspired by, and thus bears many similarities to, it. Rosemann et al. ( 24 )found that structural complexity and a higher playing tempo decreased the pianists’ EHS. However, a 30 minutes rehearsal period affected the pianists’ EHS only minimally. After the practice period, only a trend for increased EHS was detected when measured in milliseconds, but not when measured in beats. However, EHS (in beats) also correlated positively with improved performance after practice. Furthermore, EHS was related to cognitive skills (visual scanning speed, concentration, selective attention, visuomotor performance). In contrast to the current study, Rosemann et al. ( 24 ) did not operationalise prior experience as an independent variable. In general, Rosemann et al. ( 24 ) concluded “that the EHS is characteristic for each musician and developed over years of practice” (p. 672). How these characteristics develop, and how different factors affect this development, has been studied considerably less ( 21 ).

Although expertise research since decades has built a strong case for the domain-specificity of cognitive and perceptual adaptations ( 16, 31 ), Dale and Green ( 4 ) argued in their recent review that playing video games (and especially action video games) is related to “global improvements in perception, attention, memory, and executive functioning” (p. 147). For example, action video game players were shown to have a significantly better visual short-term memory ( 1, 19 )and a more flexible working memory that allows them to actively update and clear irrelevant information from it ( 3 ). Furthermore, playing action video games seems to improve perceptual processing speed ( 6, 13, 17 ), perceptual decision making ( 15 ) and the ability to track multiple objects simultaneously ( 29 ). Interestingly, research on gaming also demonstrated that action video players have a larger Useful Field of View (UFOV) and that this effect can be found on nonvideo game players after relatively short period of training ( 5, 9, 14, 32 ). Therefore it seems reasonable to expect that also eye-hand span, i.e., “the distance that the eyes are ahead of hand in playing” position ( 30 , p. 143) , is related to both prior video gaming experience and performance in platformer video games.

The aim of the present study was to investigate how prior gaming experience, cognitive skills, task difficulty and practice are related with eye-hand span and performance in the context of video gaming and early skill development. Moreover, as the design and research questions are informed by earlier research in the domain of music (most notably Rosemann et al. 24 ), a second aim is to study and discuss how the afore-mentioned perceptual and cognitive skills manifest themselves in a domain that has both notable differences and similarities to music.

The main research questions are as follows:

(1) How do a short practice period and task difficulty influence performance and eye-hand span in a platformer video game?

(2) Does eye-hand span correlate with video game performance?

(3) Are performance and eye-hand span related to prior gaming experience and cognitive skills?

## Methods

### Participants

The 33 voluntary participants (19 female, 14 male) were young adults between 20 and 35 years of age (M = 24.44 years, SD = 3.04 years). However, one male participant was excluded from the analysis due to the fact that he had played the stimulus game extensively prior to the experiment and was clearly an outlier both in terms of performance and eye-hand span. Participants’ general, self-reported, video game experience (see table 1) varied between zero and 11520 hours (Md = 264 hours, M = 2350 hours, SD = 3447 hours). Informed consent was obtained from each participant, and the participants were free to withdraw from the study at any time.

**Table 1.** Self-reported video game experience (hours)

		N	%
Video game experience	0	7	21.9
	< 2000	14	43.8
	< 4000	3	9.4
	< 6000	2	6.3
	< 8000	2	6.3
	< 10000	3	9.4
	> 10000	1	3.1
	Total	32	100.0

### Materials

**Figure 1.**
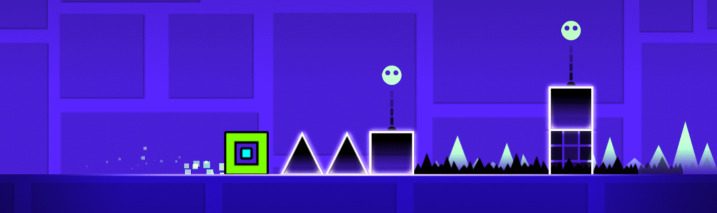
Geometry Dash video game. Player controls the green square.

The study was conducted in the context of the video game Geometry Dash (RobTop Games). The aim of the game is to navigate a small square through a series of obstacles by jumping over them (see figure 1). The only control is tapping the Spacebar key, which causes the square to jump vertically. Horizontally, the square stays in the same constant location in relation to the screen. As the game progresses, the obstacles become more complex and difficult to get through and require exact timing and multiple jumps in quick succession. In this experiment, the participants played only the levels 1 and 2, which are graded as easy by the game developer. However, the game is relatively fast-paced (speed is constant in, and between, each level). The oncoming obstacles move approximately at the speed of 880 pixels (px), or 250 mm, per second. Consequently, each obstacle moves across the whole screen in less than two seconds.

In the normal mode of the game, the better one performs, the longer each game lasts. Therefore, performance was measured by calculating the mean time (milliseconds) for all played games in each performance test phase. However, the first two games of the pre-test were excluded from the data analysis to give the participant time to familiarize themselves with the simple game mechanics.

Participants’ prior video gaming experience was operationalised by asking the participants about the number of years they have played video games and about the average amount of hours they spend playing video games per day/week/month (the participants were allowed to choose the time period that was easiest for them to assess). These numbers were then multiplied into a variable “video game experience”.

Furthermore, the participants’ cognitive skills were assessed by using the D2 Concentration Endurance Test ( 2 ) and the Trail Making Test ( 23 ). The D2 is a paper-and-pencil test of visual scanning speed, concentration and selective attention. It consists of 14 lines, each comprised of 47 characters, for a total of 658 characters. The lines are made of characters “p” and “d” with one to four vertical dashes around it. The participants are given 20 seconds to scan each line and cross out every letter "d" with two vertical dashes around it. The test was scored for both accuracy and speed. TMT is a paper-and-pencil test of visual attention and task switching. It consists of two parts. In part A the participant is instructed to connect 25 ascending numbers, and in part B the participant is instructed to connect 25 numbers and alphabet letters in ascending, alternating order. Both parts A and B are intended to be completed as quickly and as accurately as possible. Whereas part A is primarily a task of visuomotor performance ( 26 ) , part B primarily measures working memory ( 25 ), mental flexibility ( 27 ), and executive functioning ( 28 ). For each part of the test the time the participants needed to complete the task was measured with a stopwatch. Therefore, better test performance is indicated by a lower value.

### Procedure

Table 2 presents the experimental procedure in chronological order. First, the participants were introduced to the aims and to the procedure of the study. In addition to the informed consent form, the participants filled in a questionnaire including demographic questions and a self-assessment of video gaming experience.

**Table 2.** The experimental procedure in chronological order

	Phase	Materials	Duration	Data
(1)	Demographic data and, assessment of prior gaming experience and of cognitive skills	Informed consent form, questionnaire, D2, TMT	10 min	Paper-and-pencil forms
(2)	Game demonstration	Game demo	2 min	
(3)	Calibration	9-point calibration	30 sec	
(4)	Pre-test	Level 1, normal mode	2 min	Eye movements, keypresses, screen recording (first two games excluded from the data)
(5)	Practice	Level 1, practice mode	2 min	
(6)	Calibration	9-point calibration	30 sec	
(7)	Post-test1	Level 1, normal mode	2 min	Eye movements, keypresses, screen recording
(8)	Calibration	9-point calibration	30 sec	
(9)	Post-test2	Level 2, normal mode	2 min	Eye movements, keypresses, screen recording

After completing the paper-and-pencil part, the participants were given a demonstration of the game, introducing them to the aim and functionality of the game. The eye-tracker was calibrated before each test phase. In the pre-test (2 min), the participants played the game (Level 1) in a normal mode, i.e. after each failure (playercontrolled square hits an obstacle) the game starts at the same level from the beginning. During the practice phase (2 min), the participants played the game in practice mode which allows them to continue onwards from the location of the failure. The practice phase was kept short as the pilot data collections showed that longer practice periods were frustrating to the participants. Finally, posttest1 (2 min) and post-test2 (2 min) were played in the normal mode with the difference that in the post-test2, the participants played the Level 2, which they had not played or seen before. Post-test2 was administered to study how task difficulty affects performance and EHS.

### Apparatus

Eye movements were recorded using a SMI 250Hz red Eye Tracker with an accuracy of 0.5 degrees and 1680 X 1050 px monitor resolution and the related Experiment Center^TM^ software (SensoMotoric Instruments GmbH, Teltow, Germany), which also recorded the keypresses and screen activities. The head distance was set to approximately 70 cm during each calibration, but no head rest was used. Eye movements were analysed using Begaze^TM^ (SensoMotoric Instruments GmbH, Teltow, Germany) software. Eye-hand span (EHS) was defined and measured for each keypress as the distance (in pixels) between the player-controlled square and eye position. Three recordings (pre-test, post-test1, and post-test2) from two different participants had unusually low eye tracking ratios (below 80 %). After these were excluded from the data, the mean tracking ratio for the remaining data was 92 % (SD = 2.5, Min = 84, Max = 96). This was considered reasonable as the ratio is for the whole recording, including the short periods before and after each experiment (10 – 15 seconds) when the experimenter was starting and closing the game that was used in the experiment.

### Analysis

The statistical analysis was carried out using the IBM SPSS Statistics 24 software. Based on the Shapiro-Wilk test (p < .05) and visual inspection of Q-Q plots, many of the variables deviated from the normal distribution (Table 3). Therefore, non-parametric statistical analyses were used. Differences in performance and EHS between the pre-test and the post-tests were analysed with the Friedman test, post hoc Wilcoxon signed-rank tests and Independent-Samples Mann Whitney U-test. The relationship between the eye-hand span, performance, cognitive skills and prior gaming experience was analysed using Spearman’s rho correlation. Bonferroni correction was used for multiple correlations. However, as this is an exploratory study, original p values are reported and values that were not statistically significant after Bonferroni correction are marked with #.

## Results

### Practice, performance and EHS

Table 3 shows the descriptive data for all measured variables. The performance difference between the pretest, post-test1 and post-test2 was statistically significant, X²(2, 30) = 48.07, p < .001.

**Table 3.** Descriptive data

	*N*	*M*	*SD*	*Min*	*Max*	*Normally distributed*
Performance (ms)						
Pre-test	32	7,754	4,161	2,723	20,096	No
Post-test1	31	11,732	5,971	3,486	24,909	Yes
Post-test2	31	4,347	1,958	1,837	11,043	No
EHS (px)						
Pre-test	31	186	78	45	421	No
Post-test1	29	192	84	51	396	Yes
Post-test2	30	147	62	34	271	Yes
Video game experience (hours)	32	2,350	3,447	0	11,520	No
D2	32	110	11	91	129	Yes
TMT	32	86	22	42	138	Yes

Wilcoxon post hoc tests showed that after the practice phase, the average performance in the post-test1 (M = 11,732 ms) was significantly higher than in the pre-test (M = 7,754 ms, p < .001), which, in turn, was higher than in the post-test2 (M = 4,347 ms, p < .001). Considering the fact that by post-test2 the participants already had more experience playing the game than in the beginning, it seems reasonable to conclude that, compared to Level 1, Level 2 (post-test2) was significantly more difficult.

The mean EHS differed between the three measurement points, X²(2, 27) = 8.30, p = .016. However, posthoc test revealed that the difference between pre-test (M = 186 px) and post-test1 (M = 192 px) was not statistically significant (p = .468). The mean EHS in post-test2 (M = 147 px) was lower than in pre-test and post-test1 (p = .003 and p = .002, respectively). The range of the mean EHS from 147 px to 192 px corresponds to 41-54 mm distance on screen, approximately 4 degrees viewing angle and latency of 170-220 milliseconds.

In summary, performance and EHS follow a similar pattern. Practice increases the mean length of played games (performance) and EHS. However, for EHS this increase was only marginal and not statistically significant. In post-test2, both performance and EHS dropped below the pre-test level.

The relationship between EHS and performance grew weaker as the experiment advanced. The correlation between EHS and performance was the strongest in the pre-test (r_s_ = .59, p < .001). After the practice period (post-test1), the correlation was noticeably lower (r_s_ = .42, p = .025). Post-test2 did not have a statistically significant correlation with the corresponding EHS. Additionally, it is noteworthy that while both measures (performance and EHS) had reasonably high correlations between different phases of the experiment, the pattern is somewhat different:

Performance measure correlations

Pre-test – Post-test1: r_s_ = .67, p < .001

Pre-test – Post-test2: r_s_ = .85, p < .001

Post-test1 – Post-test2: r_s_ = .71, p < .001

EHS measure correlations

Pre-test – Post-test1: r_s_ = .68, p < .001

Pre-test – Post-test2: r_s_ = .54, p = .002

Post-test1 – Post-test2: r_s_ = .42, p = .030 #

Pre-test performance had the strongest correlation with post-test2, i.e., between measures where the participants were faced with a new level. The high correlations between performance measures indicate that performance differences between the participants stayed relatively stable. For EHS, the correlation was strongest between pre-test and post-test1 in which participants played the same level. Therefore, it seems that, compared to performance, the EHS differences between test phases are less stable and depend more on the task difficulty.

### The role of cognitive skills and of prior gaming experience

Neither the prior video gaming experience, the D2 Concentration Endurance Test nor the Trail Making Test (TMT) had any statistically significant correlations with any of the EHS measures. However, prior video gaming experience correlated with performance (pre-test r_s_ = .61, p < .001; post-test1 r_s_ = .39, p = .031 #; post-test2 r_s_ = .54, p = .002). The relationship between prior gaming experience and performance was strongest when the participants were playing a level that was new to them. Furthermore, D2 accuracy score correlated with performance in post-test2 (r_s_ = 39, p = .032 #) and TMT (part B) correlated negatively with pre-test (r_s_ = -.39, p = .026 #) and post-test2 (r_s_ = -.45, p = .011) performance. The correlation is negative as the TMT test is scored using the time it took the participants to finish the tasks.

Finally, to study if the cognitive skills and prior gaming experience are related to effectiveness of practice, changes in EHS and performance between pre-test and post-test1 were calculated by subtracting the post-test1 values from pre-test values (EHS and performance). While there were no statistically significant correlations between changes in performance (pre-test to post-test1), cognitive skills and video game experience, prior video game experience correlated negatively with changes in EHS (pre-test to post-test1, r_s_ = -.43, p = .020). This indicates that for more experienced video game players, the EHS was less likely to increase as a result of practice. A further analysis was conducted by dividing the participants into groups based on whether their EHS increased (n = 14) or decreased (n = 15). Mann-Whitney U-test showed that the groups did not differ in terms of video game experience or increase in performance.

## Discussion

The aim of the present study was to investigate the relationship between EHS, performance, practice, task difficulty, cognitive skills and prior experience in playing a platformer video game. EHS predicted participants’ initial performance, but this effect diminished after practice. Moreover, it seems that, instead of practice, EHS is more strongly associated with task difficulty. In comparison, practice had a strong effect on performance. Prior gaming experience and cognitive skills were not related to the size of EHS or changes in it. However, prior gaming experience improved performance (especially) when the participants were playing a level that was new to them (pre-test and post-test2). Yet, the effect was smaller after practice (post-test1). Concentration endurance (D2, accuracy) was positively related to performance in the last phase of the experiment and the Trail Making Test (TMT, part B), that measures working memory, mental flexibility and executive functioning, correlated with pre-test and post-test2 performance.

In terms of EHS, the current results converge with results obtained earlier in the domain of music ( 24 ). EHS seems to be characteristic for each individual and is not effected considerably by a short practice period. In addition, EHS is strongly related to task difficulty. However, Rosemann et al. ( 24 ) studied participants who had years of practice in the domain. The present study extends these conclusions to novices who encountered a task that was completely new to them. Still, past research on EHS and its relation to competence or experience and task difficulty is inconclusive ( 30, 10, 22 ). Therefore, to conclude whether extended practice affects EHS would require further longitudinal investigations with longer practice periods (cf. 20 ).

In addition to similarities, there are interesting differences between the results of the current study and the study conducted by Rosemann et al. ( 24 ). Despite their main conclusion, Rosemann et al. found that the practice period had a small effect on EHS (in milliseconds). In contrast, the present study found no practice effect on EHS. If individually characteristic EHS is a result of extensive domain-specific practice, one would expect that, in comparison to novices, EHS of participants who have years of domain-specific experience would be more resistant to change. Furthermore, while the musicians’ EHS (in beats) was related to better performance after practice, in the current study EHS predicted, mainly, the initial performance before practice. One possible interpretation is that musicians who improved their performance after practice were able to use their domainspecific knowledge during the practice period to partly memorize the musical structures and therefore to increase their information buffers ( 10 ). Such affordances are not available to novices.

The questions whether video gaming as an activity is related to more general improvements in perceptual skills and whether EHS is a valid measure of these skills remain open. Regarding video gaming and general perceptual skills, the current data showed no relationship between prior video gaming experience and EHS (cf. 4 ). There are at least two possible reasons for this. First, it could be due to small sample size with only few experienced video gamers. Second, it is plausible that EHS does not capture the perceptual skills video gaming, and its highly varied visual and motor tasks, develop (cf. 1, 6, 5, 9, 13, 14, 17, 19, 29, 32 ). Moreover, the relationship between EHS and performance seemed to diminish with practice. In comparison, prior video gaming experience correlated especially with pre-test and post-test2. This indicates that as one acquires experience, general perceptual skills become less important than domain-specific experience ( 16 ). Yet, it is interesting that EHS was a strong predictor of initial performance. Similarly, in studies that contrasted non-action video game players to action video game players, the instruments that were typically used to measure participants’ general visual skills were “contextfree”, i.e., they were not related to a task the participants are familiar with and therefore completely new to the participants ( 4 ). This may have implications for research on how general perceptual skills enable people to adapt to new situations.

Finally, the cognitive skills that were assessed in the current study were not related to the participants’ EHS. While Rosemann et al. ( 24 ) found a significant positive correlation between EHS and the D2 Concentration Endurance Test and a negative trend for the Trail Making Test (TMT), in the current data the same tests correlated with performance. As the results indicated, the participants with better concentration endurance (D2, accuracy) were able to keep their performance level higher in the last phase of the experiment. Therefore, D2 seems to be a valid predictor of performance in tasks that require relatively long periods of intense concentration. Furthermore, the correlations between performance in pre-test, posttest2 and TMT (part B) indicate that performance in this particular platformer game is related to working memory, mental flexibility or executive functioning (cf. 25, 27, 28 ). It is, however, plausible that these results are only applicable to situations in which one is required to adapt to a new visuo-motor task. For example, in their study of pianists’ sight-reading skill, Kopiez and Lee ( 18 ) did not find a connection between working memory and sight-reading performance. On the other hand, this could be, as they point out, due to the fact that their measure of working memory was not a spatialfigural task and, thus, did not have close relevance to sight-reading.

It should be noted that although the present study has many similarities to the Rosemann et al. ( 24 ) study, crucial differences exist concerning participants, task (static versus dynamic or moving visual stimulus), domain, and experiment. Therefore, the results are complementary, not necessarily directly comparable. In the present study, the participants were novices in playing this particular game. While many of them had had fairly extensive experience in playing video games, the task they faced in the study was a new one to them. This limits the interpretation of the present results to the early development of skills and EHS. Yet, based on their self-reported video game experience, some of the participants could (almost) be considered as experts in video gaming. Therefore, the fact that experienced video game players were better at adapting to new situations (pre-test and post-test2) begins to unravel how domain experience affects learning in adjacent domains ( 12 ). Moreover, compared to the Rosemann et al. ( 24 ) study, the task of playing a platformer game is considerably different to playing music. While in the present experiment the participants were required to perform only a very simple motor output (press key to jump), playing music is a much more complex task which requires a higher level of domain-specific knowledge and skills. Therefore, one should be careful in generalising the results of the present study to domains that require more complex expertise. Furthermore, in the current study, the practice period in the experiment was very short. Consequently, it is impossible to say how EHS and performance would develop after a more extensive training, and whether the correlations between EHS and performance would remain at a similar pattern. However, the short practice period did have a strong effect on performance. If EHS and skill development are closely connected, the effect should have also appeared for the EHS. Additionally, compared to many studies that have been conducted in the domain of music, the current study had a considerably larger sample size (cf. 30 : eight participants; 10 : eight participants; 24 : nine participants), which increases the ability to detect weaker signals and to reduce the effects of individual variation.

The present study extends the research on EHS to a new domain, video gaming, and offers insights into the early development of EHS and performance and how these are related practice, task difficulty, cognitive skills and prior experience. The results highlight the need for further studies on the dynamic nature of skill development in visual domains.

### Ethics and Conflict of Interest

The authors declare that the contents of the article are in agreement with the ethics described in http://biblio.unibe.ch/portale/elibrary/BOP/jemr/ethics.html and that there is no conflict of interest regarding the publication of this paper.
